# Traumatic Brain Injury and Blood–Brain Barrier (BBB): Underlying Pathophysiological Mechanisms and the Influence of Cigarette Smoking as a Premorbid Condition

**DOI:** 10.3390/ijms21082721

**Published:** 2020-04-14

**Authors:** Farzane Sivandzade, Faleh Alqahtani, Luca Cucullo

**Affiliations:** 1 Department of Pharmaceutical Sciences, Texas Tech University Health Sciences Center, Amarillo, TX 79106, USA; farzane.sivandzade@ttuhsc.edu; 2 Department of Pharmacology and Toxicology, College of Pharmacy, King Saud University, Riyadh 11451, Saudi Arabia; afaleh@KSU.EDU.SA; 3 Center for Blood-Brain Barrier Research, Texas Tech University Health Sciences Center, Amarillo, TX 79106, USA

**Keywords:** traumatic brain injury, blood–brain barrier, oxidative stress, cigarette smoke, neuroinflammation, excitotoxicity

## Abstract

Traumatic brain injury (TBI) is among the most pressing global health issues and prevalent causes of cerebrovascular and neurological disorders all over the world. In addition to the brain injury, TBI may also alter the systemic immune response. Thus, TBI patients become vulnerable to infections, have worse neurological outcomes, and exhibit a higher rate of mortality and morbidity. It is well established that brain injury leads to impairments of the blood–brain barrier (BBB) integrity and function, contributing to the loss of neural tissue and affecting the response to neuroprotective drugs. Thus, stabilization/protection of the BBB after TBI could be a promising strategy to limit neuronal inflammation, secondary brain damage, and acute neurodegeneration. Herein, we present a review highlighting the significant post-traumatic effects of TBI on the cerebrovascular system. These include the loss of BBB integrity and selective permeability, impact on BBB transport mechanisms, post-traumatic cerebral edema formation, and significant pathophysiological factors that may further exacerbate post-traumatic BBB dysfunctions. Furthermore, we discuss the post-traumatic impacts of chronic smoking, which has been recently shown to act as a premorbid condition that impairs post-TBI recovery. Indeed, understanding the underlying molecular mechanisms associated with TBI damage is essential to better understand the pathogenesis and progression of post-traumatic secondary brain injury and the development of targeted treatments to improve outcomes and speed up the recovery process. Therapies aimed at restoring/protecting the BBB may reduce the post-traumatic burden of TBI by minimizing the impairment of brain homeostasis and help to restore an optimal microenvironment to support neuronal repair.

## 1. Introduction

**Figure 1:**
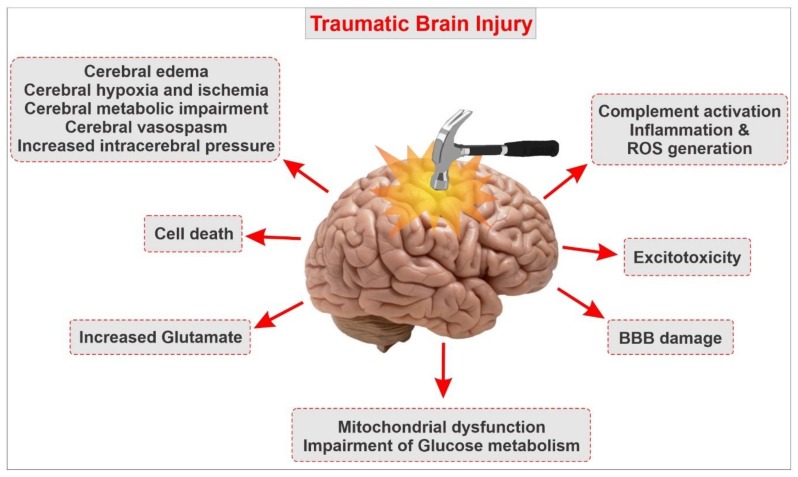
Simple schematic outlining the pathophysiological responses following traumatic brain injury and the complex outburst of secondary impairments. Note that secondary injury processes of traumatic brain injury (TBI) include blood–brain barrier (BBB) disruption, neuroinflammation, excitotoxicity, metabolic impairments, apoptosis, oxidative stress, ischemia, and others. Associated with BBB impairment, microglial and astrocyte activation, leukocyte infiltration, and upregulation of pro-inflammatory cytokines are characteristic of the neuroinflammatory response of TBI.

Traumatic brain injury (TBI) is defined as an insult to the brain caused by a direct or indirect external mechanical force. TBI has long been among the foremost leading causes of death and disability in the United States, thus becoming a serious public health concern in modern society [1,2,3,4,5,6,7]. According to the Centers for Disease Control and Prevention (CDC), every year, about 2.5 million people in the U.S. seek emergency care for TBI secondary to motor vehicle accidents, falls, assaults, sports-related events, and other mechanisms. In addition, every year, more than 5.3 million Americans live with a lifelong disability caused by TBI [8,9,10]. In those that survive, the effects of TBI can cause emotional, physiological, cognitive, motor, and behavioral impairments ranging from mild to severe [5,11,12,13,14]. Mild traumatic brain injury (mTBI) accounts for over 80% of head injuries [1]. mTBI typically results in transient symptoms, including sensitivity to light and sound, headache, vision impairment, difficulties with cognition, and balance. The severity of TBI is classified into three degrees (depending on the length of unconsciousness following the head injuries), including mild TBI (loss of consciousness ≈ 15 s), moderate TBI (loss of consciousness of several minutes), and severe TBI (loss of consciousness ≥ 1 h) [15]. Approximately 20–40% of patients die after a severe TBI due to brain injury or secondary complications, and those that survive often have reduced life expectancies, chronic neurological disabilities, pituitary dysfunction, and cognitive and psychological disorders, including depression and aggression [1,15,16,17,18]. In fact, after a moderate or severe TBI, most patients require hospitalization for medical management. During the post-hospital recovery phase, they often deal with reduced cognitive abilities, anxiety and depression disorder, and impaired balance and coordination. Needless to say that these post-traumatic effects burden the patient with a higher risk of re-hospitalization, as well as an additional economic burden for the individual and his/her family and reduced quality of life [17,18]. Researchers in the field have been focusing on several aspects of TBI, including the physical characteristics of the trauma and the lapse time between trauma and the initial onset of neuropathologies [19]. The development of TBI is divided into two general stages: primary (immediate) injury and secondary (delayed) injury [2,20]. The primary trauma encompasses all acute pathological changes, such as shearing injuries, contusions, and hematomas [2,21]. Secondary changes, including the formation of cerebral edema, oxidative stress (OS), inflammation, excitotoxicity, imbalanced calcium homeostasis, enhanced vascular permeability, and blood–brain barrier (BBB) impairment often occur following vascular and parenchymal damage in the brain. Secondary injury events can significantly exacerbate post-traumatic brain injury and worsen clinical outcomes [22,23,24] (see also Figure 1).

Concerning the involvement of the BBB, substantial pieces of evidence are suggesting that TBI impairs the BBB, and BBB damage is implicated in the loss of neurons, altered brain function, and possibly the response to therapy. Post-traumatic dysfunction of the BBB is one of the significant factors determining the progression of injury and affecting the time course and the extent of neuronal repair [25]. This review covers the pathogenesis of TBI and encompasses the major pathophysiological factors that promote post-traumatic dysfunction of the BBB and its involvement in the onset of post-TBI neuropathologies. We also provide a brief review of the current treatment used to alleviate the burden of TBI.

## 2. The Blood–Brain Barrier Interface

The BBB is a dynamic functional interface between the blood and the central nervous system (CNS), which allows nutrients, essential amino acids, ions, etc. to transport between the peripheral circulation and the brain. At the same time, it inhibits many pathogens and toxic compounds from entering the brain [26,27,28,29]. The primary functions of the BBB are to maintain the homeostasis in the brain and protect the brain from potentially endogenous and xenobiotics, leading to optimal neuronal activity [3,15]. Generally, the BBB is formed by the brain endothelial cells [30]. The junctions between endothelial cells are tightly connected through the adherens junction (AJ) proteins such as cadherins and tight junctional proteins such as occludin and claudins [31]. Besides, astrocytes, microglia, and pericytes are also critical for the normal function of the BBB and the phenotype of brain endothelial cells so that they contribute to the formation and maintenance, selectivity, and specificity of the BBB [19,32]. Glial and endothelial cells functionally interact with each other in a paracrine manner contributing to the integrity of the BBB [30]. The efflux transporters present on the endothelial cells, such as the P-glycoprotein (Pgp), multidrug-resistant protein (MRP), and breast-cancer-resistance protein (BCRP) support the selective function of the BBB by actively moving various lipophilic drugs out of brain endothelial cells. Together, the physical and metabolic barrier of the BBB plays a crucial role in maintaining brain homeostasis.

## 3. Chronic Smoking: A Major Comorbid Factor for BBB Dysfunction and Significant Neurological Disorders

Tobacco smoke (TS) is a diverse mixture of over 4700 toxic compounds, including carcinogens and mutagenic chemicals, as well as stable and unstable free radicals and reactive oxygen species (ROS). Chronic smoking is one of the leading preventable causes of morbidity and mortality that affects the function of almost every organ of the body, giving rise to a range of illnesses that reduce life expectancy in smokers [33,34]. TS is responsible for approximately 6 million deaths per year in the world and more than 480,000 deaths each year just in the United States (US). Smoking has been strongly associated with enhanced the risk of stroke and other cerebrovascular and neurological disorders such as Alzheimer’s, multiple sclerosis, and vascular dementia [35]. The mechanisms through which TS promotes the onset and progression of various cerebrovascular and neurodegenerative diseases are multifaceted. They include OS, BBB dysfunction, inflammation, alteration of cellular redox metabolic activities, and the activation of immune responses [36,37]. There is numerous evidence suggesting the pivotal role of oxidative stress in endothelial dysfunction and following BBB damage in the cerebrovascular level [38]. The exposure of tobacco smoke even at non-toxic concentration induces a robust inflammatory response in cells that influences the cerebrovascular endothelium and circulating immune cells [39]. The inflammatory response and reduced cerebral blood flow, which are both are common in chronic smokers, result in exaggerated the damage of BBB, suggesting its contribution toward cerebrovascular disease and ischemic stroke [40]. It has been claimed by various studies that smoking increases the risk of neuroinflammatory disorders, i.e., Alzheimer’s disease, and multiple sclerosis [40]. A previous study showed that chronic prenatal exposure to nicotine could induce the unusual discharge of neurochemicals, followed by several pathological consequences in children. The study also demonstrated that nicotine causes an abnormal differentiation of neuronal cells, decreases synaptic activity, and promotes apoptosis, thus leading to abnormal brain development. Tobacco smoke may provoke the expression of several adhesion molecules and the release of tumor necrosis factor-alpha (TNF-α), interleukin-6 (IL-6), and matrix metalloproteinase-2 (MMP-2). Altogether, these are essential in the regulation of leukocytes movement across the endothelium into adjacent tissue and contribute toward perivascular inflammation. Altered BBB integrity and increased atherogenesis may eventually lead to ischemic insult. The pro-inflammatory role of TS might be the result of its impact on the transcription of genes that are involved in the pathogenesis of atherosclerosis and modulate the inflammatory reaction at the BBB level. TS can cause the significant upregulation of various transcription factors, which can play an essential role in the gene expression of cytokines, chemokines, and different adhesion molecules, i.e., E-selectin and Intercellular Adhesion Molecule-1 (ICAM-1). TS can also upregulate Signal Transducer and Activator of Transcription-3 (STAT3), which is an angiogenesis modulator that acts through the IL-6/STAT3 signaling pathway [41]. STAT3 links extracellular signals to transcriptional control of proliferation and cycle progression [42]. Furthermore, TS may also contribute to the upregulation of Apo-lipoprotein E (ApoE) and Serum Amyloid A1 (SAA1) genes. ApoE plays a role in the regulation the lipoprotein metabolism, and it is known to associate with elevated cholesterol and risk of atherosclerosis and ischemic stroke. The transcriptional product of the SAA1 gene is serum amyloid, which is a potent chemoattractant that modulates tissue infiltration and the adhesion of monocytes and leukocytes [43]. In addition, amyloid in brain vessels can enhance BBB permeability [44]. All these events result in the loss of BBB integrity during ischemic insults and contribute toward the development of cerebrovascular diseases. TS can also downregulate claudin-5 expression, which is an essential interendothelial tight junction protein involved in maintaining the BBB integrity and function. There is considerable controversy regarding the influence of premorbid and comorbid conditions that commonly accompany TBI such as cigarette smoking on the expected rate and extent of recovery from TBI [10]. Indeed, considering the influence of premorbid and comorbid conditions may lead to spurious conclusions about the mechanisms underlying the pathophysiology of the TBI [10].

## 4. Pathophysiology and Underlying Causes of TBI

The pathophysiology of TBI can be divided into primary and secondary injury mechanisms. The primary mechanical injury is due to the physical trauma from a direct head impact. It may result in intracranial and extracranial hemorrhage, tissue destruction, and axonal shearing following damage to the blood vessels, brain tissue, and the BBB [15,45]. Within a variable period (days to months and sometimes even years) after the onset of the initial injury, secondary injury usually occurs. The pathogenic events unfolding during the onset of the secondary damage ultimately compromise cell and tissue viability at the metabolic and molecular levels [23]. Cerebral edema formation and the increase of intracranial pressure are other critical factors that are prodromal to secondary brain injury [15]. There are several neurotransmitters, biochemical mediators, cytokines, and genetic changes that play a pivotal role in the molecular mechanisms of tissue injury after TBI [46]. This pro-inflammatory environment (promoting OS and the increased expression of endothelial cell adhesion molecules) facilitates the activation and the influx of immune cells into the brain parenchyma, thus determining the progression of injury, including excitotoxicity and neuronal loss [25,47,48]. Although the primary brain injury is the initial pathogenic factor, the secondary brain injury is generally more severe and complex and promotes the expansion of the initial mechanical brain injury in surrounding healthy tissue [20,24,49,50]. Several studies reported that TBI chronically leads to the onset of various cerebrovascular and neurodegenerative disorders such as Alzheimer’s disease, chronic traumatic encephalopathy, and epilepsy as well as other long-term problems, including the loss of executive function, inappropriate social behavior, and cognitive disabilities [51,52,53].

## 5. TBI and Breakdown of the BBB

BBB disruption is a major pathophysiological feature of TBI and is associated with neuroinflammatory events contributing to brain edema and cell death [2]. Due to the highly heterogeneous characteristic of the brain, in response to a direct impact or external forces applied to the head, different brain structures are subject to different acceleration rates, resulting in the generation of considerable tensile, shear, and compressive forces within the brain.

**Figure 2:**
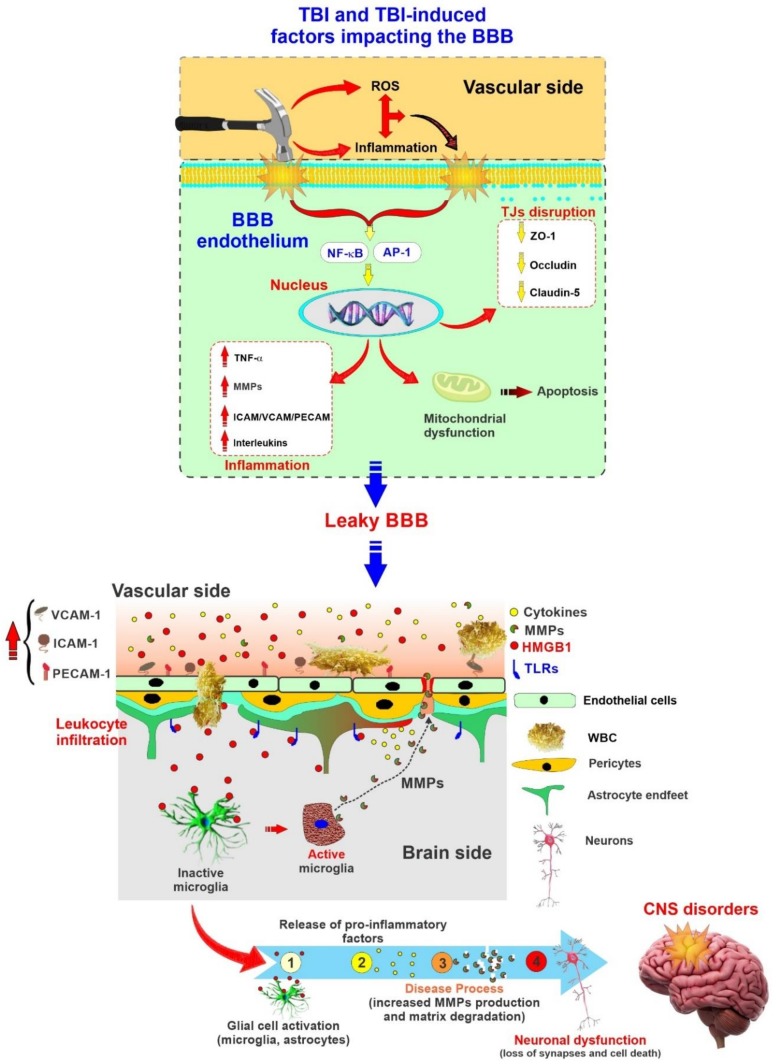
Schematic illustration depicting TBI and TBI-depending factors impacting the BBB and the onset of secondary brain injuries. Representation of BBB as a source and target of neuroinflammation in TBI. Note that inflammation and reactive oxygen species (ROS) generation associated with TBI can further impact the BBB in addition to mechanical trauma. The loss of BBB integrity further promotes neuronal damage and the onset of central nervous system (CNS) disorders.

Both astrocytes and microglia can rapidly respond to injury by increasing the production of multiple factors that may have a significant effect on BBB function [30]. The BBB permeability is mainly modulated by the expression of tight junctional proteins on endothelial cells [2]. However, the primary injury of TBI leads to damaged endothelial cells and a loss of blood flow followed by disrupted tight junctional proteins and the basal membrane, thus leading to a loss of barrier integrity and subsequent elevated permeability [25] (see also Figure 2). BBB breakdown not only triggers leukocyte recruitment and the migration of inflammatory cells activating astrocytes, but it also causes the release of pro-inflammatory cytokines, cytotoxic proteases, and reactive oxygen species (ROS) to activate microglia, affecting neuronal activity [15]. A physical barrier termed “glial scar” forms around the damaged area to protect the surrounding intact neural tissue from the destructive immunoresponse and prevent the spread of inflammation to neighboring neurons and undamaged areas.

Moreover, local inflammation occurs, expanding the site of injury and exacerbating the damage [3]. The glial scar encloses an area containing inhibitory molecules that impede the regrowth of neurons and block the repair of the BBB [4,15]. Furthermore, disruption of the BBB after TBI promotes the activation of the coagulation cascade, resulting in the formation of an intravascular blood clot and ischemia [2]. In addition, the reduced integrity of the BBB enables the efflux of plasma proteins into the extravascular space. This latter efflux ultimately induces neuroinflammation by promoting the activation of transforming growth factor β (TGF-β) [54].

These studies suggest that there is a strong association between the degree of BBB disruption and onset of neuroinflammatory processes caused by circulating immune cells and the influx of neutrophils into the damaged area of the CNS [2]. This set of events promotes the onset of a vicious cycle, which helps further damage to the BBB. On the other hand, a leakier BBB may facilitate the efflux of water from the brain parenchyma, thus reducing post-TBI cerebral edema [2]. In fact, researchers have demonstrated that transient and size-selective modulation in the BBB paradoxically enhances the movement of water from the brain parenchyma to the blood vessels, leading to decreased brain swelling [55].

## 6. Post-TBI Cell Death Mechanisms

Mechanisms of neuronal cell death post-TBI have been mainly categorized into necrosis and apoptosis, while there are at least a dozen mechanisms of neuronal death including intrinsic and extrinsic apoptosis, oncosis, necroptosis, parthanatos, ferroptosis, sarmoptosis, autophagic cell death, autosis, autolysis, paraptosis, pyroptosis, phagoptosis, and mitochondrial permeability transition [56]. Necrosis is defined as a passive process characterized by the loss of ionic balance and membrane integrity of the cell, leading to cell and intracellular organelle swelling [46]. On the other hand, apoptosis is an energy-consuming process characterized by condensation and fragmentation of the cytoplasm and nucleus with the maintenance of organelle structure, which occurs as programmed cell death and a controlled part of an organism’s growth or development [21,46]. TBI promotes the upregulation of many cell-cycle activators such as c-myc, cyclins, and cyclin-dependent kinases, and the downregulation of cell-cycle inhibitors. Moreover, after TBI, caspase-dependent pathways (which play a significant role in cell death) are activated and result in an imbalance between proapoptotic (Bax and Bad) and anti-apoptotic (Blc-2 and Bcl-xL) molecules enhancing cell death [57,58]. Besides, apoptosis-inducing factor (AIF) released into the cytosol, which modulates cell death, causes condensation of chromatin in the periphery of the nucleus along with DNA fragmentation. Studies have shown that the release of AIF is dependent on Poly(ADP-Ribose) Polymerase 1 (PARP-1), causing depletion of cytosolic NAD+ and, consequently, mitochondrial dysfunction and outer membrane permeabilization [46]. It accounts for why PARP-1 inhibition after TBI has proven to be neuroprotective. Autophagic programmed cell death (which is the natural, regulated mechanism of the cell that removes unnecessary or dysfunctional components) involves the lysosomal degradation of organelles and proteins. Autophagy is often the dominant mechanism for programmed cell death in conditions of mitochondrial permeabilization and inhibition of caspases [21].

On the other hand, neuronal cell death is a much more complicated process involving autophagy and paraptosis as well. Paraptosis has a non-apoptotic morphology and is regulated by genes [59,60]. Neuronal cell death is categorized in physiologic and excitotoxic [46]. Physiologic cell death refers to a developmental death due to injuries such as ethanol or TBI and is characterized by initial mitochondrial swelling followed by vacuolization of the endoplasmic reticulum after rupture of the nuclear membrane. Excitotoxic cell death (often seen in a few hours after TBI) refers to the rapid swelling and rupture of organelles along with the clumping of chromatin in the center of the nucleus [61,62].

Autophagy is one of the main types of neuronal death after TBI [63]. Autophagy flux occurs with the fusion of the autophagosome with the lysosome to form autolysosomes, which degrades the cytoplasmic organoids [64]. Three general methods to detect autophagic flux are Microtubule-associated protein 1A/1B-light chain 3 (LC3) turnover, protein p62 degradation, and tandem fluorescent tagged LC3 (tfLC3) assay [65]. Autophagy has a dual role in TBI, depending on its flux [66]. In mild cases, autophagy flux is increased, which is expected to be beneficial for cell survival. Conversely, flux is decreased in moderate to severe cases, which leads to neuronal death and aggravated pathological phenotypes [66]. Increased LC3-II and autophagosomes are observed in the experimental weight drop injury model of TBI [67], and caloric restriction after mild TBI results in increased Beclin 1, LC3, and mTOR [68]. TBI could inhibit PI3K/AKT/mTOR pathway [69], NRF2/ARE pathway [50], TLR4/NF-κB pathway [70], and activate FoxO3a [71] and Drp1 [72] proteins, which are in the upstream of autophagy and their regulation by TBI may promote autophagosome formation and cause BBB disruption [66].

## 7. Excitotoxicity

Cellular excitotoxicity is a pathological process by which neuron cells are damaged by excessive neurotransmitters stimulation such as glutamate and other excitatory factors. Cellular excitotoxicity, which is a crucial mediator in the pathophysiology of TBI, primarily occurs as a result of upregulated N-methyl-D-aspartic acid (NMDA) and α-amino-3-hydroxy5-methyl-4-isoxazole -propionic acid (AMPA) receptors [73]. Raised glutamate released from parenchymal brain cells activates the AMPA receptors, prompting the receptor-associated ion channels to open, thus upregulating the influx of sodium and calcium ions [2,74]. Glutamate levels are the highest immediately after TBI, and these heightened levels are sustained for 24–48 h due to the disruption of the BBB [75]. In addition, there is some evidence that glutamate promotes ROS production [76,77].

There are some other molecules, including TGF-β, vascular endothelial growth factor (VEGF), and matrix metalloproteinases (MMPs), which become abnormally elevated in the brain following TBI, thus contributing to BBB impairment and the loss of barrier integrity [2]. The damage to the BBB will eventually facilitate the development of cerebral edema and the onset of other post-TBI secondary injuries [2]. Specifically, recent studies have revealed the increased synthesis and elevated levels of MMPs as well as a reduced expression of MMPs inhibitors and endogenous MMPs regulators in plasma and the cerebrospinal fluid (CSF) of brain tissue from TBI patients and animal models of TBI.

Moreover, the upregulation of VEGF, as a major regulator of endothelial cell proliferation, angiogenesis, and vascular permeability as well as the downregulation of claudin-5 expression was correlated to BBB dysfunction [26,33,78,79].

TGF-β, as a molecule with a pivotal role in cell proliferation and differentiation, gets released in large amounts from platelets after vascular wall damage. Several studies have shown that there is an increase in the expression of both TGF-β and TGF-β receptors on vascular endothelium post-TBI. There are conflicting studies regarding the positive or negative impact of TGF-β on BBB permeability, so some believe TGF-β plays a role in maintaining BBB integrity through stabilizing endothelial cell and pericyte interaction via N-cadherin. Other studies instead suggest that TGF-β derived-tyrosine phosphorylation reduces claudin-5 and VE-cadherin expression [80,81].

## 8. Neuroinflammation

Neuroinflammatory responses to injury have a significant pathophysiological role in the development of post-TBI secondary brain damage (although its role in primary injury is limited). Immediately after the impact, the mechanical disruption of the BBB causes the extravasation of red blood cells, accompanied by a limited influx of leukocytes due to the rapid activation of the coagulation cascade. The process results in a significant reduction in blood flow in affected brain tissue [30]. Neuroinflammation involves the influx of leukocytes into the injured brain parenchyma penetrating across the BBB. This is followed by the influx of neutrophils, monocytes, and lymphocytes within a relatively short period of time (hours to days) post-TBI [82,83,84]. During this critical time frame, the production of inflammatory mediators, including cytokines (such as IL-6, IL-10, TNF-α, and IL-1β) increases. Following the cytokines’ upregulation, cell adhesion molecules expressed on the surface of the cerebrovascular endothelium also increase. All of these processes eventually lead to the influx of inflammatory cells from the blood into the brain to initiate a host of restorative processes, including neurogenesis, synaptogenesis, oligodendrogenesis, and angiogenesis taking place in the brain as the spontaneous functional recovery after TBI [85,86,87,88,89,90]. Another factor of relevance is that the activation of microglial cells not only causes the amplification of the inflammatory response but also ROS production along with neurotoxic molecules. The result is the onset of other secondary mechanisms of cell death [83].

As mentioned previously, cytokines are pro-inflammatory mediators that include interferons, interleukins, and chemokines secreted from immune cells [91]. Based on recent in vivo studies, the synthesis of pro-inflammatory cytokines is rapidly upregulated in rodent models of TBI [30]. Among the major cytokines, TNF-α and IL-1β play a vital role in exacerbating tissue damage; thus, there is a dose–response correlation between the cytokines expression level and mortality, including intracranial pressure and multiorgan failure [46]. While TNF-α increases as early as one hour after TBI, IL-1β upregulates gradually to reach a peak at 6–8 h post-TBI. Pro-inflammatory cytokines also have multiple effects on the BBB, where they promote loss of barrier integrity, downregulation and altered distribution of tight junctional proteins, and ROS production [25,92]. Moreover, further details have revealed that TNF-α enhances the formation of actin stress fibers, which is followed by cell retraction and formation of intercellular gaps. In addition to the detrimental effect on BBB viability, the most critical role of these pro-inflammatory mediators is inducing the synthesis of chemokine as well as the expression of cell adhesion molecules on the surface of the endothelial layer. In several studies on human brain microvascular endothelial cells, it has been demonstrated that cells exposed to TNF-α or IL-1β promoted the expression of E-selectin, Intercellular Adhesion Molecule 1 (ICAM1), and vascular cell adhesion molecule-1 (VCAM1) on the cell surface [30]. Furthermore, the rapid induction of endothelial expression of E-selectin and an upregulated expression of ICAM1 post-TBI have been reported in both clinical and animal studies and have been positively correlated with increasing severity of the injury and worsening neurological outcome.

Nuclear factor kappa-light chain-enhancer of activated B cells (NF-ĸB) is a protein complex that controls pro-inflammatory cytokine production in most cell types, including neurons, astrocytes, microglia, oligodendrocytes, and endothelial cells of neurovascular and cerebrovascular units [21]. Recent studies have demonstrated a critical physiological role for the NF-ĸB signaling pathway in the central nervous system, serving crucial functions in cellular responses to neuronal injury [93,94,95]. It is well established that post-TBI leads to the activation of NF-κB in glial cells and neurons in the same brain region undergoing atrophy, which is associated with inflammatory processes. Moreover, it is reported that repression of the NF-κB inhibitor system in an experimental model of TBI promoted neuronal cell death, worsens the neurological outcome, and increased the post-TBI mortality rate [96].

## 9. Cerebral Edema Formation

Cerebral edema is among the very significant secondary injury consequences of TBI and the leading cause of death in more than half of all deaths after severe TBI [15]. Understanding the development of cerebral edema is crucial because it considerably affects the high morbidity and mortality after TBI [2]. Post-traumatic cerebral edema caused the expansion of brain volume against an enclosed skull and raised intracranial pressure inside the unyielding cranial cavity, causing herniation and a reduction in cerebral perfusion pressure, promoting cerebral ischemia [97]. According to valid studies, cerebral edema results from a combination of endothelial cell damage, tight junction disruption, and abnormal transcellular transport due to vessel damage resulting in interstitial accumulation of plasma-derived, osmotically active molecules followed by water [30,55]. Cerebral edema could also be caused by changes in cell metabolism and the failure of membrane-associated pumps and ion transporters, resulting in the cellular accumulation of osmotically active molecules followed by water [2].

## 10. Oxidative Stress and Influence of Cigarette Smoking on the Pathophysiology of TBI

Excessive ROS production following cell damage, neuronal cell death, and brain dysfunction are the results of several secondary biochemical and metabolic changes in the cells [24]. Oxidative stress, which results from the unhindered generation of ROS, has been known as the major pathophysiological mechanism responsible for secondary injury post-TBI [98]. Post-traumatic oxidative stress leads to the peroxidation of membrane polyunsaturated fatty acids, protein carbonylation, and DNA oxidation through ROS, which may affect the BBB permeability and fluidity, leading to membrane damage and eventual apoptosis and tissue necrosis [99,100,101]. Antioxidant mechanisms, including superoxide dehydrogenase, catalase, and peroxidases, are often detrimentally affected by TBI, which leads to increased oxidative injury [102]. There is evidence that the interstitial level of hydroxyl radicals increases rapidly after TBI, and it may play a significant role in the lipid peroxidation of the membrane, eventually causing highly active aldehydes, such as 4-hydroxynonenal (4-HNE) [30]. Several in vitro and in vivo models of the BBB have shown that 4-HNE significantly increases BBB permeability. At the same time, the administration of lipid peroxidation inhibitor reduces the post-traumatic increase in BBB permeability. Normal BBB function is highly dependent on the ability of BBB to protect themselves from noxious effects of ROS through endogenous molecules such as glutathione (GSH). The pharmacological depletion of GSH significantly increases the paracellular BBB to low molecular weight substances. The exposure of the BBB to a mixture of ROS predominantly containing superoxide anion radicals (produced as a result of NADPH oxidase (Nox) upregulation), hydroxyl radicals, and hydrogen peroxide was shown to rapidly increase the BBB permeability. The loss of BBB integrity was associated with the redistribution and degradation of tight junctional proteins, as well as DNA degradation and lipid peroxidation [17,101]. It is well known that nitric oxide rapidly reacts with superoxide anion radical, resulting in a variety of free radicals and eventually oxidative stress. Although the cerebrovascular endothelium itself produces nitric oxide in small quantities, the exposure to moderate to the high amount of nitric oxide significantly promotes the paracellular permeability of BBB [30]. Moreover, oxidative stress may play a significant role in promoting post-traumatic neuroinflammation as a result of increased adhesion and migration of monocytic cells as well as the expression of intercellular adhesion molecules across the endothelial monolayers. Furthermore, bradykinin is known as a promoter of ROS production so that it activates phospholipase A2, which induces Nox activity, further leading to ROS production [25]. It has been proven Nox inhibitors are able to reduce inflammation, neuronal degeneration, oxidative stress, and cerebral edema post-TBI [103,104].

According to recent studies, NRF2 plays a significant neuroprotective role in TBI and other neurodegenerative disorders so that NRF2 activation counteracts TBI-induced OS, loss of BBB integrity, etc. [20,22]. Unsurprisingly, impairments of the NRF2–ARE pathway leading to the reduced activity of this protective system can lead to more extensive post-TBI tissue damage, thus aggravating the secondary injury and worsening the outcome. Accordingly, the upregulation of NRF2 expression could be exploited to reduce post-TBI outcomes [80]. TBI-induced brain damage provides a viable strategy to treat post-traumatic brain injuries, improve clinical outcomes, and reduce the risk of additional neurological disorders through the reduction of oxidative stress and post-traumatic inflammatory responses [105,106,107].

## 11. Current Treatments of TBI

Millions of people in the United States have been suffering through chronic symptoms of TBI [108]. While TBI is a prominent health crisis that faced global public health leading to very high mortality and morbidity, unfortunately, there has been a lack of effective therapeutic options for the management of TBI cases. However, some nutritional components and therapeutic agents have currently been utilized for the treatment of the symptoms at earlier stages of TBI.

Elevated calcium levels play an essential role in the cascade of cellular damage after TBI [109]. Thus, the use of calcium channel blockers has been proposed for the prevention of or treatment after cerebral brain injury, causing secondary brain damage due to a reduction in blood flow [110]. **Nimodipine** and **ziconotide** are drugs that can block L- and N-type calcium channels, respectively. Based on recent studies, the administration of nimodipine regulates brain perfusion and prevents further neuronal damage, resulting in improved symptoms of TBI patients who have subarachnoid hemorrhage [110]. Similarly, in a rat model, it has been shown that if ziconotide was administered within 15 min to 6 h of TBI, it leads to improved mitochondrial function as well as motor and cognitive activities [111,112]. It is noteworthy that in spite of the beneficial effect of nimodipine on brain injury patients with subarachnoid hemorrhage, the increase in adverse reactions may mean that the drug is harmful to some patients [110].

Brain swelling in TBI patients may cause dangerous pressure on the brain (increased intracranial pressure (ICP)). **Mannitol,** which is a naturally occurring alcohol found in fruits and vegetables and used as an osmotic diuretic, has demonstrated a beneficial role in reducing ICP and inflammatory response as well as improving blood perfusion in a dose-dependent manner [109,113]. In contrast, it is revealed that although mannitol reverses the swelling at first, its prolonged use can eventually worsen the pressure [114]. Therefore, additional evidence is necessary to show whether administrating mannitol leads to the improvement of post-TBI outcomes.

**Amantadine** is one of the most prescribed medications for patients with prolonged disorders of consciousness after TBI. Based on several studies, amantadine accelerates the pace of functional recovery during active treatment in patients with post-TBI disorders of consciousness [115]. Besides, in the acute phase of TBI, neurons can be protected from glutamatergic excitation by administering amantadine as its action as an NMDA antagonist in frontal lobes and may improve cognition [116]. Although amantadine showed generally improved conditions in TBI patients, in several studies, no differences were found in the rate of cognitive improvement between subjects given amantadine versus those given placebo.

Neurons majorly lose their function, leading to death during secondary brain injury, which has a series of consecutive pathological processes resulting from oxidative stress [49,117]. The metabolism of biological macromolecules produces excessive ROS that intensely damages neurons after TBI [24]. NRF2 as a critical regulator protein that prevents oxidative stress damage due to injury and/or inflammation after TBI [118]. Thus, its activation may attenuate post-TBI pathophysiologic phenomena, i.e., neuronal apoptosis cerebral edema and cognitive deficits. Indeed, recent promising studies encourage the utilization of NRF2 as a targeted therapy for the patients suffering from TBI.

**Curcumin**, a polyphenol derived from Curcuma longa rhizomes, has been examined for the underlying mechanism behind its neuroprotective role in secondary brain damage after TBI [22,119,120]. The beneficial effects of curcumin are not only is due to its ability to cross the BBB, but also it has been linked to the activation of NRF2 as well as superoxide dismutase and glutathione peroxidase [21,121,122,123,124,125,126]. Recent studies have also revealed that curcumin lessened brain edema and oxidative damage by enhancing NRF2 expression and [22,127,128] antioxidant enzymes, i.e., Nqo1, Hmox1 Gclm, and Gclc, as well as down-regulating NF-kB. Additionally, several studies have indicated that curcumin could promote membrane and energy homeostasis and influence synaptic plasticity following TBI [129,130].

**Sulforaphane** (SFN), a well-known NRF2 promoter/activator, is an organic isothiocyanate naturally found in cruciferous plants such as broccoli and cabbage. SFN has been shown to possess neuroprotective properties in vitro as well as in vivo by activating the NRF2–KEAP1 pathway and enhancing the corresponding protein synthesis [122,131,132,133,134,135,136,137,138,139]. Several studies revealed that in addition to the activation of NRF2, SFN could independently inhibit NF-kB activity. SFN has been shown to inhibit TNF-alpha-induced NF-kB activation through the inhibition of IkB-α phosphorylation, degradation, and p65 nuclear translocation, thus preventing cell apoptosis [140]. Moreover, SFN was shown to be able to block the direct interaction between NF-kB and its consensus sequence, suppressing its pro-inflammatory activities in T cells [141].

**Metformin** (MF) is a biguanide oral anti-hyperglycemic agent derived from the plant Galega Officinalis and used to treat patients with type-2 diabetes (non-insulin dependent). MF has also been shown to attenuate BBB disruption via AMPK-dependent and independent (NRF2 antioxidant pathway) mechanisms [142,143]. Recent studies revealed that NRF2 plays a protective role in TS-induced cerebrovascular/BBB impairments by normalizing TJs proteins expression, restoring the BBB integrity, and decreasing oxidative stress and inflammation, which all leads to a reduced burden of TS cerebrovascular toxicity [144]. Not surprisingly, MF has also been shown to concurrently inhibit NF-κB activation, thus preventing the cytokine-induced expression of pro-inflammatory and adhesion molecules in vascular endothelial cells [145,146,147,148]. These studies highlight the additional MF therapeutic potential outside the treatment of diabetes [149,150]. These findings highlight the other MF therapeutic potential outside the treatment of diabetes for the treatment of cerebrovascular and neuroinflammatory disorders associated with oxidative stress and inflammation, primarily due to its dual effect as an NRF2 activator and NF-κB inhibitor [144,151,152,153].

**Ramelteon** is a melatonin receptor agonist that is used to treat sleep disturbance in patients with TBI. In recent studies on TBI, ramelteon has demonstrated antioxidant and anti-inflammatory properties [154,155]. Timed ramelteon treatment appeared to protect the brain after TBI by preventing inflammation. This effect seems to be mediated by a drug-dependent increase of anti-inflammatory cytokines and the activation of the NRF2–ARE signaling pathway [154,155]. Thus, it seems that ramelteon could be considered as a potential chronotherapeutic strategy for treating TBI.

A huge number of drugs with anti-inflammatory action have been investigated in both preclinical and clinical studies. Mostly, these drugs are categorized into either anti-inflammatory inducers or inflammatory inhibitors [156]. Interestingly, the efficacy of anti-inflammatory drugs is directly assessed through the reduced number and activation state of inflammatory cells as well as changes in the levels of anti-inflammatory mediators. In an outstanding review paper, Bergold has provided a comprehensive review on the results of both preclinical studies and clinical trials of anti-inflammatory drugs [156].

## 12. Conclusions

The consequences of TBI are a growing concern in the United States. Despite vast studies, the mechanisms and therapeutic strategies to alleviate the impacts of TBI have not been fully understood. Excitotoxicity, oxidative stress, cerebral edema formation, neuroinflammation, and cytokine are prominent mechanisms of cell damage and death post-TBI. Several novel promising strategies to treat TBI are successfully targeting the stabilization of BBB integrity. The safekeeping of BBB function could have a significant therapeutic relevance to improve TBI outcomes and reduce secondary injury. Indeed, regulating post-traumatic BBB dysfunction would play an active role in the reduction of morbidity and mortality from TBI. This is even more relevant in patients with premorbid conditions (such as chronic smoking), which have detrimental effects on BBB function and immune responses. Although the therapeutic window of BBB regulation after TBI remains unknown, further elucidation of the dynamics of BBB dysfunction after TBI would provide significant knowledge for novel specific therapies, the selection of therapeutic agents, and the timing of treatment. Future investigations might determine how to effectively engage the cerebrovascular endothelium to produce neuroprotective growth factors that can act on nearby neurons. As the understanding of the complex cellular and molecular interactions within the gliovascular unit grows, it is likely that more opportunities for interventions that are directed at the BBB will arise.

## Abbreviations

4-HNE	4-hydroxynonenal
AJ	Adherens junctions
AMPA	α-amino-3-hydroxy5-methyl-4-isoxazole-propionic acid
ApoE	Apo-lipoprotein E
BBB	Blood–Brain Barrier
BCRP	Breast-cancer-resistance protein
CNS	Central nervous system
CDC	lefts for Disease Control and Prevention
FTC	Federal Trade Control
ICAM1	Intercellular adhesion molecule 1
IL-6	Interleukin-6
MF	Metformin
MMP-2	Matrix metalloproteinase-2
MRP	Multidrug-resistant protein
NAD(P)H	Quinone reductase I
NF-ĸB	Nuclear factor kappa-light chain-enhancer of activated B cells
NMDA	N-methyl-D-aspartic acid
NRF2	Nuclear factor erythroid 2-related factor
OS	Oxidative stress
PARP1	Poly(ADP-Ribose) polymerase 1
PECAM-1	Platelet Endothelial Cell Adhesion Molecule-1
Pgp	P-glycoprotein
ROS	Reactive oxygen species
SAA1	Serum Amyloid A1
SCSM	Single cigarette smoking machine
STAT3	Signal Transducer and Activator Of Transcription-3
SFN	Sulforaphane
TBI	Traumatic Brain Injury
TGF-β	transforming growth factor β
TJ	Tight Junction
TNF-α	tumor necrosis factor-alpha
TS	Tobacco smoke
VCAM1	Vascular cell adhesion molecule-1
VEGF	vascular endothelial growth factor
ZO-1	Zonula occludens-1
